# Efficacy and application of a novel topical anaesthetic wound formulation for treating cattle with Foot‐and‐Mouth disease: A field trial in Cameroon

**DOI:** 10.1111/tbed.13923

**Published:** 2020-12-05

**Authors:** Sevidzem S. Lendzele, Jacques F. Mavoungou, Kong A. Burinyuy, Koumba A. Armel, Simon J. Dickmu, James R. Young, Peter C. Thomson, Peter A. Windsor

**Affiliations:** ^1^ Institut de recherche en Ecologie Tropicale (IRET‐CENAREST) Libreville Gabon; ^2^ Ecole Doctorale des Grandes Ecoles (EDGE) Libreville Gabon; ^3^ Université des Sciences et Techniques (USTM) Franceville Gabon; ^4^ School of Veterinary Science and Medicine University of Ngaoundéré Ngaoundéré Cameroon; ^5^ The National Veterinary Laboratory (LANAVET) Garoua North Cameroon; ^6^ Sydney School of Veterinary Sciences The University of Sydney Camden NSW Australia; ^7^ School of Life and Environmental Sciences The University of Sydney Camden NSW Australia

**Keywords:** animal welfare, Cameroon, cattle, foot‐and‐mouth disease, therapeutic efficacy

## Abstract

Recently, a wound dressing formulation, (Tri‐Solfen®, Medical Ethics Pty Ltd, Australia; TS) registered for use in ruminant husbandry in Australia, was registered for Foot‐and‐Mouth Disease (FMD) therapy in large ruminants in Laos, following clinical observations of improved welfare and healing following treatment of FMD lesions. In November 2019, an FMD outbreak in Cameroon provided an opportunity for a field trial, comparing clinical responses and recoveries to treatments on a sample of cattle (*n* = 36) comprising three equal groups of animals (*n* = 12), comparing responses to three treatments: (i) the application to lesions of TS, (ii) the administration of parenteral oxytetraycline commonly used for FMD in Cameroon; and (iii) an untreated control group (C). Appetite scores, lesion healing scores, and changes in dimensions of lesions, were recorded over a 15‐day study period. Cattle treated with TS achieved both superior appetite and lesion healing scores with more rapid reduction in dimensions of lesions than other groups. Farmer observations indicated the TS treatment group had a more rapid return to eating with cessation of excessive salivation, and more rapid return of mobility (walking) with absence of overt lameness. The findings indicate that although mortality is usually low in FMD outbreaks, the disease is a debilitating and painful disorder with negative animal welfare impacts that should be addressed. All farmers expressed their desire that the product be made available for use in their region and modelling indicates that TS therapy imposes no additional financial burden on farmers, with the treatment likely to be provided at a similar or reduced cost to current treatment choices. As use of antibiotics for treatment of a viral disease potentially increases pressures for development of antimicrobial resistance and residues in the food chain, TS as an alternative non‐antimicrobial therapy should be promoted for wider use in FMD outbreaks.

## 1 INTRODUCTION

Cameroon, with a population of almost 28 million people, is located in central Africa, bordering the Gulf of Guinea. It is a key transit link, sharing borders with six countries: Nigeria, Chad, Central African Republic, Republic of Congo and Equatorial Guinea. The gross domestic product (GDP) per capita of Cameroon is estimated at USD 3,700 (CIA, 2017). The approximately 7.1 million cattle in Cameroon are susceptible to regular outbreaks from Foot‐and‐Mouth Disease (FMD), and endemic disease in much of Africa. With no preventive control programmes in place and no access to commercial FMD vaccination, farmers are focused on treatment choices for affected cattle, with antibiotics and/or traditional therapies commonly used and prolonged periods for animals to recover.

FMD is a most important global viral pathogen of artiodactyl farmed and wildlife animals. The disease is characterized by lesions in and around the mouth and feet (Fakhrul‐Islam et al., 2016). Globally, there are seven pools of circulating FMD viruses recognized. Each pool represents independently circulating and evolving FMD virus (FMDV) genotypes. Within the pools, cycles of emergence and spread occur that usually affect multiple countries in the region. In the absence of specific and laboratory‐confirmed reports, it should be assumed that the prevalent serotypes are continuously circulating in parts of the pool area and would be detected if sufficient surveillance was in place. Cameroon sits in the West/Central African region designated as Pool 5. FMD causes huge economic losses in Cameroon, with estimation of the total annual cost of FMD management at USD112 million (FAO, 2015).

Of the seven FMDV serotypes, four (O, A, SAT 1 & SAT2) occur in Cameroon (Ehizibolo et al., 2019; Lendzele, Abdoulmoumini, et al., 2019; Ludi et al., 2016). There is no mass vaccination programme for FMD and no commercial FMD vaccines are available in Cameroon (Bertram et al., 2018). A pilot trial using commercial trivalent vaccine (Aftovax®) was conducted in 2015 in Ngaoundere, with observations that clinical infection of FMD appeared to have been prevented, although subclinical persistent infection occurred lasting approximately one month, as confirmed by field observations (Bertram et al., 2018). Cattle owners in Cameroon routinely manage the disease using a range of therapies, including antibiotics, anti‐inflammatory preparations and traditional formulations (Lendzele, Marvoungou et al., 2019).

In the absence of strategic preventive control programmes, there are urgent needs for alternative FMD management options for endemic FMD‐infected countries, including Cameroon. With affordable and efficacious vaccine candidates for managing FMD unlikely in the near future, the review and rationalizing of FMD therapies are advisable. Several topical treatments with ethno‐veterinary and recognized veterinary pharmaceuticals have been examined in FMD endemic settings in Africa (Al‐Lethie et al., 2018; Fakhrul‐Islam et al., 2016; Gakuya et al., 2011; Misk et al., 2015). However, the widespread use of parenteral antibiotics for FMD globally, including numerous countries in Africa and Cameroon in particular, presents antimicrobial resistance (AMR) and potential food safety residue risks that need to be addressed.

Recently, an Australian wound dressing formulation, Tri‐Solfen® (Medical Ethics Pty Ltd, Australia; TS) registered for use in cattle and small ruminant husbandry in Australia and New Zealand, was registered for FMD therapy in large ruminants in Laos in south east Asia (Windsor et al., 2020). The wound and lesion dressing formulation contains two local anaesthetics (lignocaine and bupivacaine), adrenalin and cetramide in a gel matrix that creates a barrier effect, numbing the pain of lesions, rapidly reducing their infectivity, and hastening healing, potentially reducing the weight loss in affected individuals (Windsor et al., 2020). If made available for purchase and administration by farmers, this product could provide a viable alternative approach for managing FMD in Cameroon, other African countries and developing countries globally. This formulation offers numerous advantages over current therapies as it provides efficacious pain relief and more rapid healing of wounds and lesions (Roberts & Windsor, 2019; Windsor et al., 2016, 2020). Further, with a pH of ~2.7, and containing the antiseptic cetrimide, it potentially has viricidal impacts and anti‐bacterial properties respectively, avoiding the need for other treatments, including antibiotics (Windsor et al., 2020).

In November 2019, TS was provided to the Cameroon research team for trials during FMD outbreaks in cattle involving the extensive smallholder cattle raising system. There are three cattle husbandry systems in Cameroon, including: (1) intensive, with animals raised in pens and supplied grass and food supplements (e.g. cotton cakes) without access to pastures and where attendance by veterinarians is common; (2) semi‐intensive, with animals kept in traditional pens that also enable them to access pastures freely; and (3) extensive, with animals pastured freely through the day, then returned to traditional pens before dusk where they spend the night, rarely receiving food supplements or attended by a veterinary technician (para‐veterinarian) or veterinarian.

This report describes a field trial conducted on extensive cattle farms in Cameroon aimed at evaluating the efficacy of this therapy for FMD lesion management, particularly for enhancing recovery and wound healing. The study compared the clinical responses from TS therapy to the most commonly used and available antimicrobial therapy currently in use, parenteral oxytetracycline. If TS is proven to be as efficacious for FMD in this current study as it appeared to be on first use in Laos as described (Windsor et al., 2020), it is likely that the product could offer an important innovation for improving FMD lesion treatment, potentially globally, with reduced animal welfare burdens, risks of AMR issues and possibly, increased transboundary disease reporting and surveillance.

## 2 MATERIALS AND METHODS

### 2.1 Ethics statement

In addition to adopting current procedures on animal and human ethics processes in Cameroon, this collaboration ensured compliance with the National Health and Medical Research Council's (NHMRC) National Statement on Ethical Conduct in Human Research (2007) and the Universities Australia Australian Code for the Responsible Conduct of Research. This included ensuring that all participants provided verbal informed consent for the administration of therapies, collection of animal samples, observations and interviews, plus participation in videos and images, where written consent was unavailable due to farmer illiteracy.

### 2.2 Trial location and design

**FIGURE 1:**
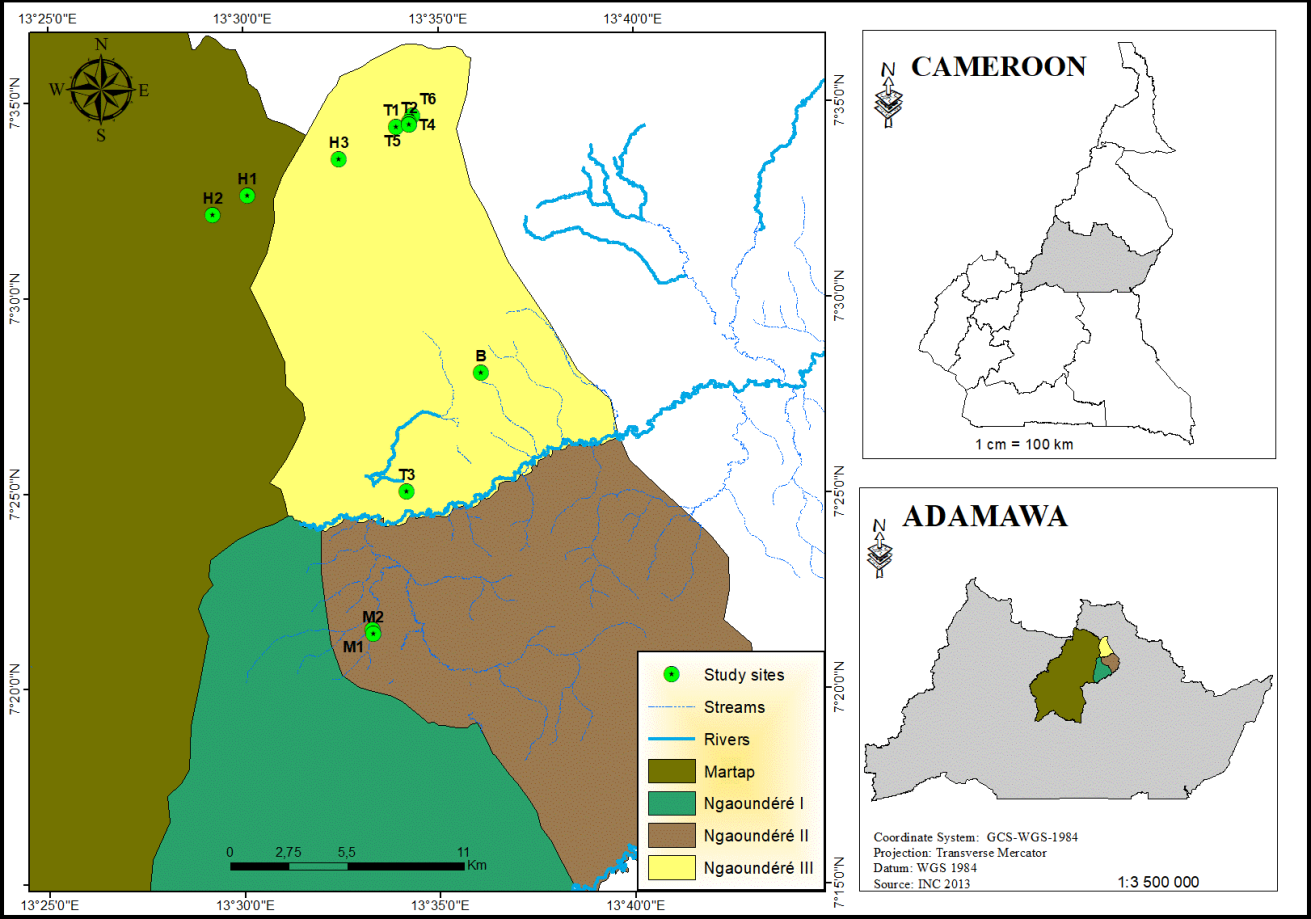
Maps showing the study sites (Ngaoundere II, Ngaoundere III and Martap). M1: Mbidjoro 1, M2: Mbidjoro 2, T1: Tchabal Baouro 1, T2: Tchabal Baouro 2, T3: Tchabal Baouro 3, T4: Tchabal 4, T5: Tchabal 5, T6: Tchabal 6, B: Borongo, H1: Horé Mayanga 1, H2: Horé Mayanga 2, H3: Horé Mayanga 3 [Colour figure can be viewed at wileyonlinelibrary.com]

This clinical trial was conducted between the 5th of November and the 5th of December 2019 in Ngaoundere II, Ngaoundere III and Martap subdivisions in the Vina Division of the Adamawa plateau. Around 40% of the cattle population of Cameroon is located in the Adamawa region (MINEPIA, 2013). Ngaoundere is the capital of the Adamawa region, with geo‐referenced coordinates between 6° 40' 0" and 7° 30' 0" north latitude and between 13° 20' 0" and 14° 10' 0" east longitude (Figure 1). An FMD outbreak in the municipalities of Ngaoundere II, Ngaoundere III and Martap was advised to the lead author by a field technician in Ngaoundere on the 4th of November 2019. The following day, the outbreak areas were accessed, confirming the typical lesions of vesicular to ulcerative glossitis and coronitis consistent with clinical signs of FMD. The treatment trials commenced immediately. Two teams of two members each were formed to enable animal treatments and follow‐up monitoring of the treated cases. Participating farmers were selected following demonstration of owner willingness to participate, and included extensive smallholder cattle farms (designated as T1, T2 etc) from the villages of Horé Mayanga, Borongo, Tchabal Baouro and Mbidjoro. The GPS data of all farms were recorded.

As Moore‐Oxy^®^ (MO; oxytetracycline HCL 5%, Hebei Kexing Pharmaceutical Co. Ltd., Shijiazhuang City, China) was the reference parenteral antibiotic commonly used by farmers to manage clinical FMD in the study area (Lendzele, Marvoungou, et al., 2019), it was decided to compare the efficacy of this formulation on the healing of FMD lesions of the coronary band with that of a single topical treatment with TS. As MO is administered intra‐muscularly and TS is administered topically, on each farm, three animals were matched by age and breed, with similar FMD clinical presentations; with one treated with MO, one with TS and the third left untreated. This created 12 sets of cattle comprising 3 animals each for this comparative clinical trial on the different farms (Table 1).

**TABLE 1:** Age and breed of cattle recruited into each treatment group

Variable	Treatment group			Overall
	Moore‐Oxy^®^	Tri‐Solfen^®^	Control	
Average age (year)	3.7	4	3.3	3.7
Age range (year)	2 to 5	2 to 8	2 to 5	2 to 8
Breed count				
Goudali	9	9	10	28
Holstein	1	1	1	3
White Fulani	1	2	1	4
Red Fulani	1	0	0	1
Total	12	12	12	36

### 2.3 Treatment applications

The clinical treatment trial was conducted with animals displaying ‘fresh’ oral and coronary band FMD lesions, observed as intact vesicles or recently ruptured vesicles. Of the 12 farmers enrolled into the 15‐day treatment trial with three matched‐cattle, each received a different treatment, with the age and breed of the cattle recruited into each treatment group presented (Table 1).

Animals treated with TS had all lesions liberally sprayed with a single topical application of up to 2 ml of the product, as per label instructions. Animals treated with MO received intra‐muscular injections of the product daily for 3 days, also at doses per label instructions (2‐4 mg/kg). Clinical response observations were initially conducted and recorded by veterinary technicians with experience of observing animals with clinical FMD and these personnel trained the farmers in how to conduct the required observations. All assessors were blind to treatment to avoid bias. The observations included the recording of any improvements in demeanour and interest and capacity of the animal to walk and eat, plus the following semi‐quantitative clinical measurements:

- coronary band lesion healing score (LHS) on a scale of 1 to 4 (adapted from Al‐Lethie et al., 2018):
- coronary band lesion size (cm): healing of coronary band lesions was measured quantitatively in the field using a 30 cm ruler, every 3 days from day zero (D0) through day 15 (D15), with lesions photographed using android mobile phones; and
- appetite score (AS) on a scale of 1 to 4, reflecting both interest in eating, noting that animals with fever generally display anorexia, and ability to eat, noting that animals with oral ulceration may not have the capacity to prehend (adapted from Al‐Lethie et al., 2018).

### 2.4 Farmer observations

The farmers were also blind to treatment and after training, were requested to provide their observations for each animal for each treatment group, with the following information recorded:

- number of days until cattle mobility (walking) returned;
- number of days cattle were not eating;
- number of days cattle continued to salivate in response to the presence of oral lesions;
- number of days cattle displayed lameness, reflecting the likely progress in healing of painful foot lesions; and
- number of days required for cattle to return to grazing on pasture.

### 2.5 Treatment cost model

The costs of treatments were estimated and used to develop a cost‐benefit model enabling comparison of therapies that could assist decisions for both individual farmers and public health policy makers on FMD outbreak management.

### 2.6 Statistical analyses

#### 2.6.1 Ordinal scale analysis

Both the lesion healing scores and the appetite scores were recorded on a four‐point ordinal scale, not a quantitative scale, so appropriate ordinal categorical methods were required for this analysis (Agresti, 2002). In addition, walking was also modelled on a three‐point ordinal scale: (1: immobile; 2: walking with difficulty; 3: walking normally). An ordinal logistic mixed model was fitted to each data set with fixed effects for Treatment, Day, Breed and Age (covariate), and a random effect for the individual Animal ID. A Treatment × Day interaction was included in each of the models, to allow for a different shaped time course for each treatment. Note that the fitted model returned a set of model‐based probabilities of obtaining each possible score (1 through 4, or 1 through 3), for the particular combination of terms in the model. The model was fitted using the clmm function in the ordinal package of R (Christensen, 2019), and probability estimates obtained using the emmeans (Lenth, 2020) and RVAideMemoire (Hervé, 2020) packages in R.

#### 2.6.2 Binary data analysis

The status of cattle being on pasture and cattle salivating (Yes = 1, No = 0) is binary outcomes, and for this, binary logistic mixed models were used to analyse these data. As above, fixed effects for Treatment, Day, Treatment × Day, Age, Breed and Sex, and a random effect for Animal ID were included in the model. The model was fitted using the glmer function in the lme4 package of R (Bates et al., 2015), and model‐based means obtained using the emmeans package.

#### 2.6.3 Quantitative data analysis

Lesion size (cm) was analysed using a linear mixed model, with fixed effects for Treatment, Day, Treatment × Day, Age, Breed and Sex, and a random effect for Animal ID. Model fitting was via the lmer function in the lme4, and model‐based means obtained using the emmeans package. Event duration data (number of days that cattle are immobile, walk with difficulty, walk normally, on pasture, and salivating) were analysed using linear models with fixed effects for Treatment, Age, Breed and Sex. Due to the positive skew, a log*_e_*(*y* + 1) transformation was applied. However, with the large number of ‘zero’ durations, hypothesis testing was conducted with permutation tests rather than *F* tests, using the aovperm function in the permuco package (Frossardm & Renaud, 2019) in R.

## 3 RESULTS

### 3.1 Lesion healing scores

**FIGURE 2:**
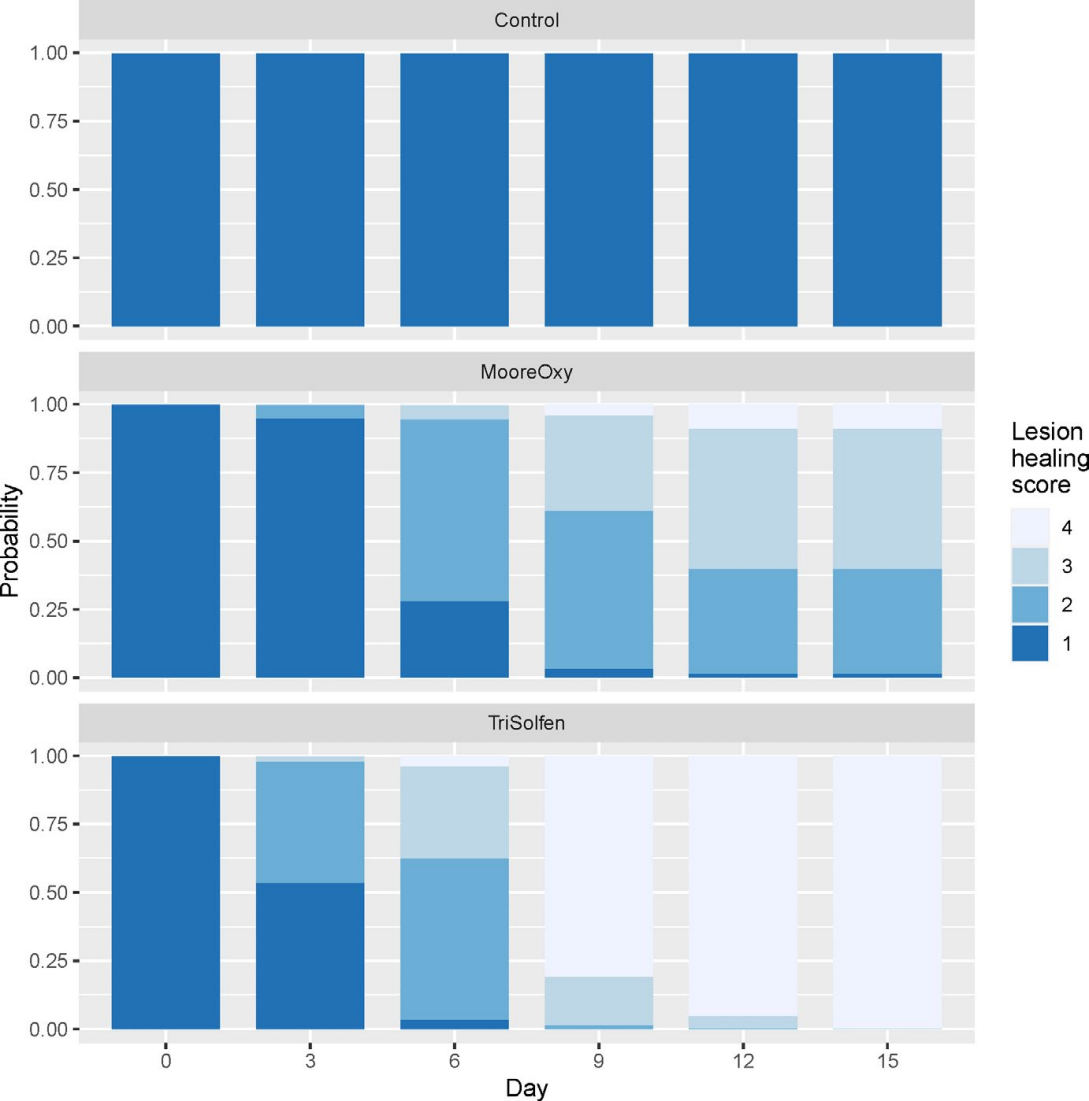
Model‐based probabilities of obtaining lesion healing scores 1 through 4 across the six study days, for the three treatment groups [Colour figure can be viewed at wileyonlinelibrary.com]

There was a highly significant Treatment × Day interaction (*p* = 3.4 × 10^–7^), indicating differing lesion healing score time courses across the three treatment groups. Model‐based lesion score probabilities are displayed (Figure 2). While control cattle maintained the presence of erosions/ulcers (a score of 1), recoveries were observed in the two treatment groups. From Day 9 onwards, cattle treated with TS had significantly higher lesion healing scores than those on MO (Day 9: *p* = .025; Day 12: *p* = .016; Day 15: *p* = .0008). None of the other terms had a significant association with lesion healing score: Age: *p* = .464; Breed: *p* = .311; Sex: *p* = .376.

### 3.2 Appetite scores

**FIGURE 3:**
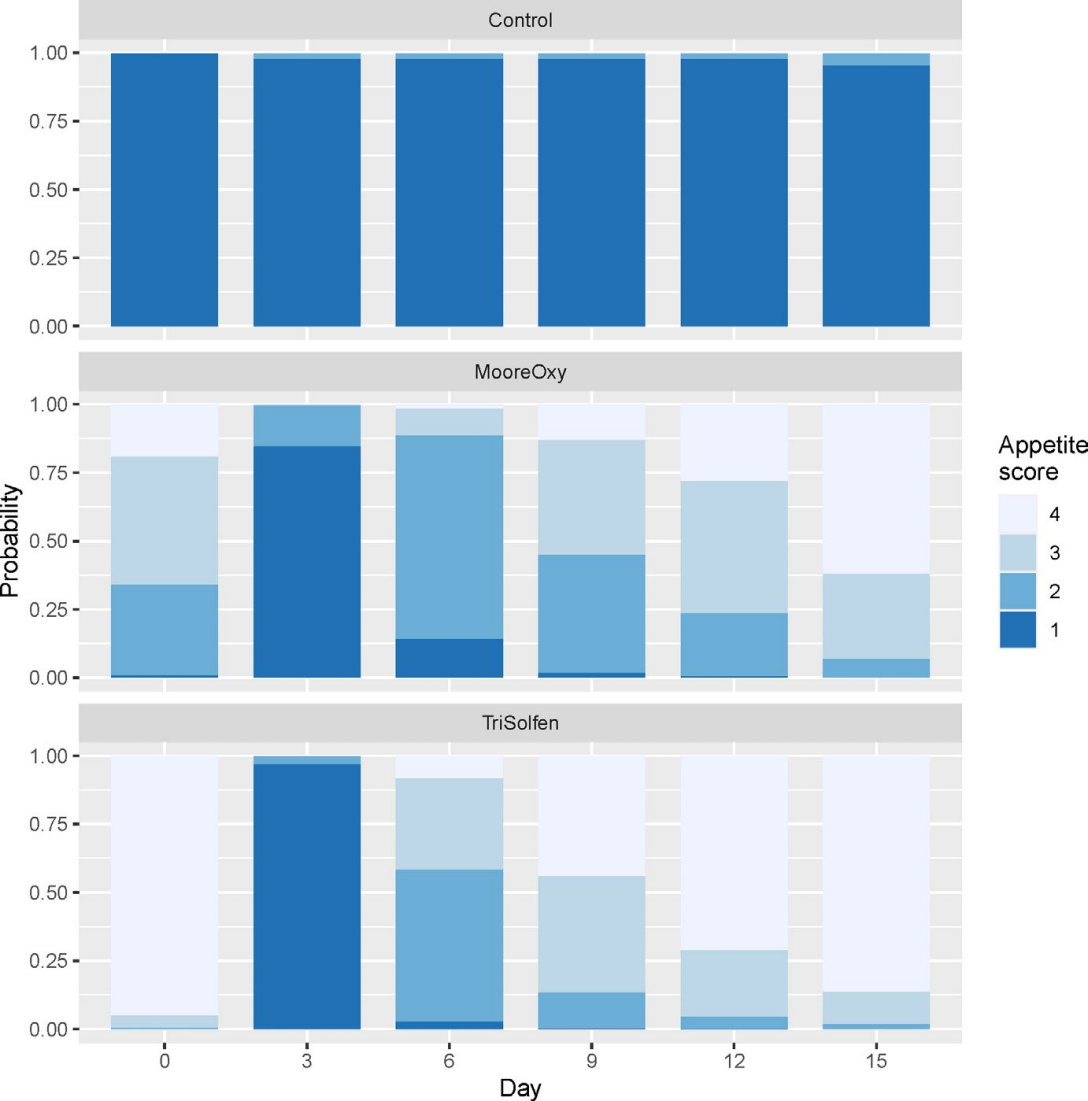
Model‐based probabilities of obtaining appetite scores 1 through 4 across the six study days, for the three treatment groups [Colour figure can be viewed at wileyonlinelibrary.com]

Again, there was a highly significant Treatment × Day interaction (*p* = 2.0 × 10^–6^) for appetite score, indicating treatment effects were changing over the study period. Model‐based appetite score probabilities are displayed (Figure 3). Most control cattle had no appetite with low scores over the duration of the study period (score of 1). However, the two treatment groups had moderate (MO) to high (TS) scores on Day 0, low on Day 3, then progressively increasing scores after that. After Day 0, there were no significant differences in scores between MO and TS‐treated cattle (all *p* > .10). Neither Age (*p* = .810), Breed (*p* = .524), nor Sex (*p* = .324) had a significant effect in appetite score.

### 3.3 Lesion size

**FIGURE 4:**
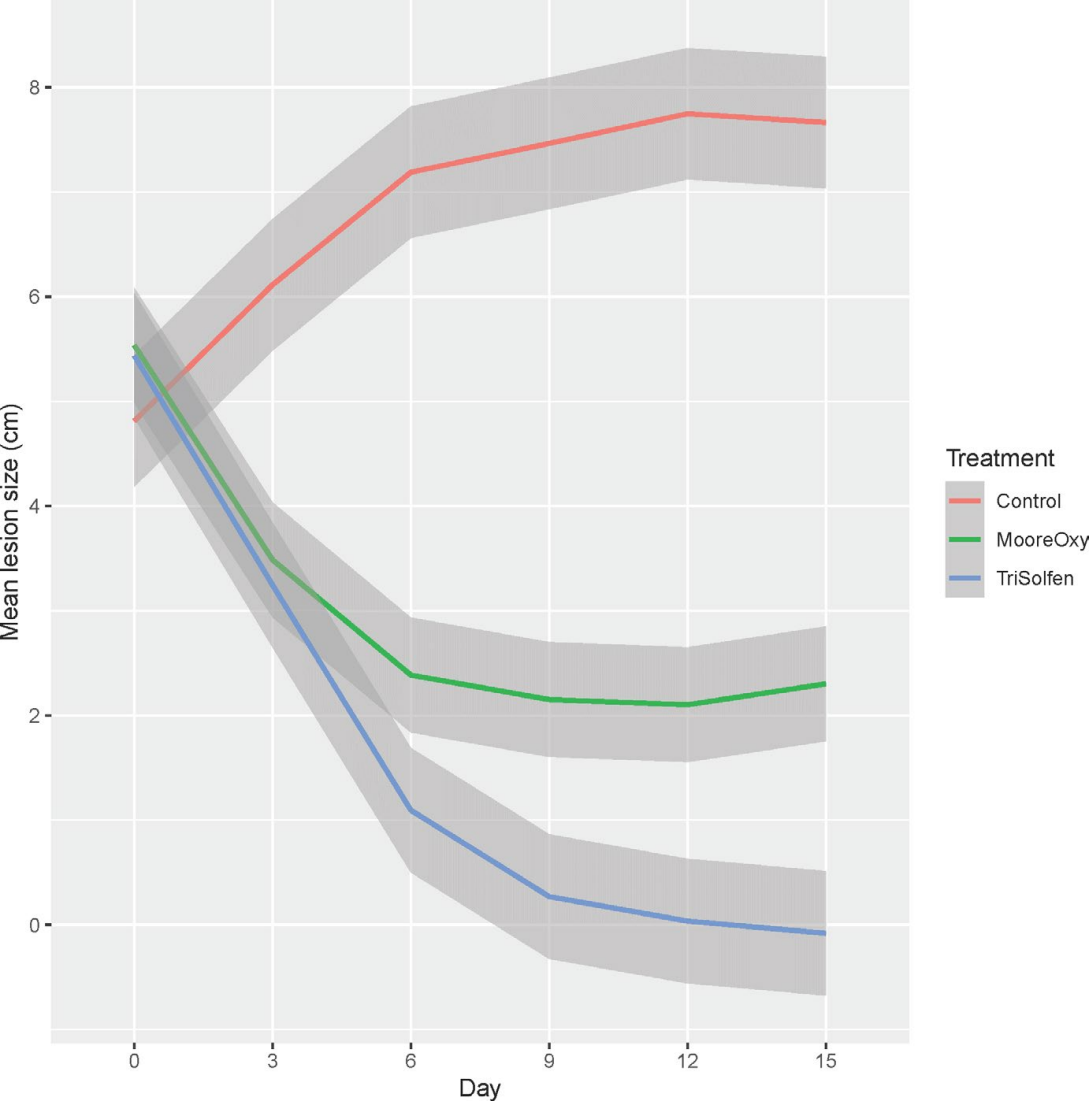
Model‐based mean lesion size (cm) across the six study days, for the three treatment groups. Shaded areas are ±1 *SE* of the mean [Colour figure can be viewed at wileyonlinelibrary.com]

There was a highly significant Treatment × Day interaction (*p* < 2 × 10^–16^) for lesion size (Figure 4), indicating different rates of healing across the three groups. Initially, there were no significant differences in mean wound sizes amongst the three groups (all *p* > .25). While mean wound size increased for Control cattle, they reduced for the two treatment groups, with those in the TS treatment reducing at a faster rate and differences were significant from Day 9, (all *p* < .01). There was no significant effect of Age (*p* = .454), Breed (*p* = .225), nor Sex (*p* = .374) on lesion size.

### 3.4 Walking

**FIGURE 5:**
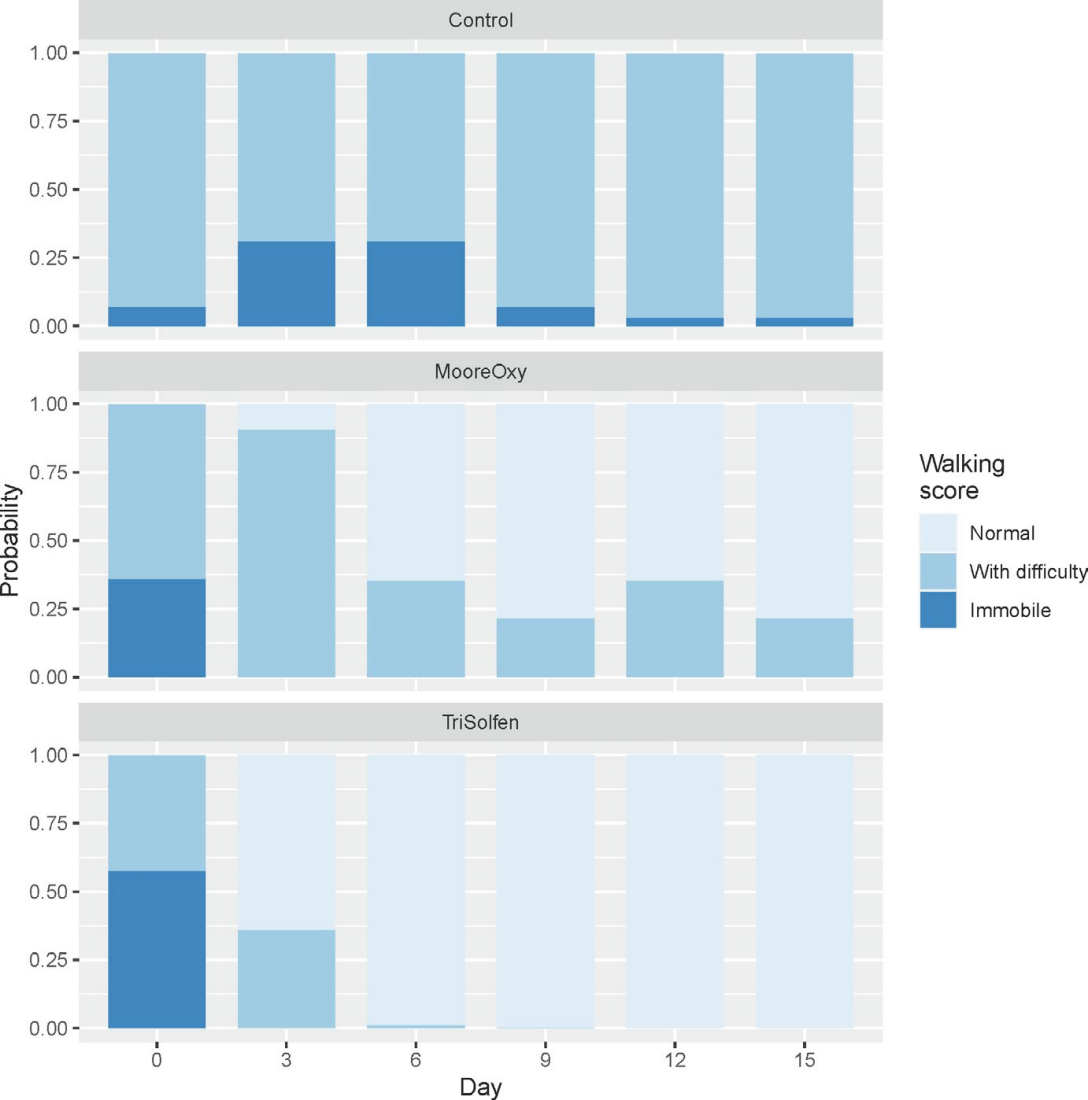
Model‐based probabilities of obtaining walking scores of ‘Immobile’, ‘With difficulty’ and ‘Normal’ across the six study days, for the three treatment groups [Colour figure can be viewed at wileyonlinelibrary.com]

When walking is considered as an ordinal score (Immobile < With difficulty < Normal), there was a highly significant Treatment × Day interaction (*p* = 1.8 × 10^–13^). Initially (Day 0), there were no differences in walking scores between the three groups (all *p* > .15) and some cattle remained ‘Immobile’ or ‘With difficulty’ throughout the study period (Figure 5). However, walking improved for the two treatment groups, particularly for the TS treatment group. However, the differences between these two groups never reached threshold significance (all *p* > .05). There were no significant effects of other factors on walking (Age: *p* = .583; Breed: *p* = .9012; Sex: *p* = .778).

An additional analysis was conducted to compare the number of days that cattle are immobile, number of days they walk with difficulty, and number of days they walk normally (Table 2). In all cases, these durations differed significantly between the three treatment groups (all *p* < .01). Control cattle had a significantly longer period of immobility compared with the two treatment groups (both *p* < .005).

**TABLE 2:** Analysis of the number of days that cattle are immobile, number of days walking with difficulty and number of days walking normally

Walking	Variable	*p*‐Value	Control	Moore‐Oxy^®^	Tri‐Solfen^®^
Immobile	Treatment	.002	2.47^A^± 1.39	0.05^B^ ± 0.35	0.00^B^ ± 0.30
	Age	.942			
	Breed	.590			
	Sex	.268			
With difficulty	Treatment	.009	8.27^A^ ± 4.95	3.49^AB^ ± 2.00	0.70^B^ ± 0.85
	Age	.471			
	Breed	.875			
	Sex	.724			
Normal	Treatment	.000	0.60^A^ ± 0.40	2.72^B^ ± 0.78	7.18^C^ ± 1.92
	Age	.221			
	Breed	.117			
	Sex	.033			

Note

Means sharing the same superscript alphabets are not significantly different (*p* > .05).

TS‐treated cattle had a significantly shorter period of walking with difficulty compared with Control cattle (*p* = .0022), and also had significantly longer period walking normally compared with both Control and MO‐treated cattle (both *p* < .005). Note that there were no effects of Age, Breed, not Sex on the three durations, with the exception of Sex on duration of normal walking (*p* = .033) with male cattle having mean duration 3.72 ± 1.14 days, compared with female duration of 1.82 days.

### 3.5 Time on pasture

**FIGURE 6:**
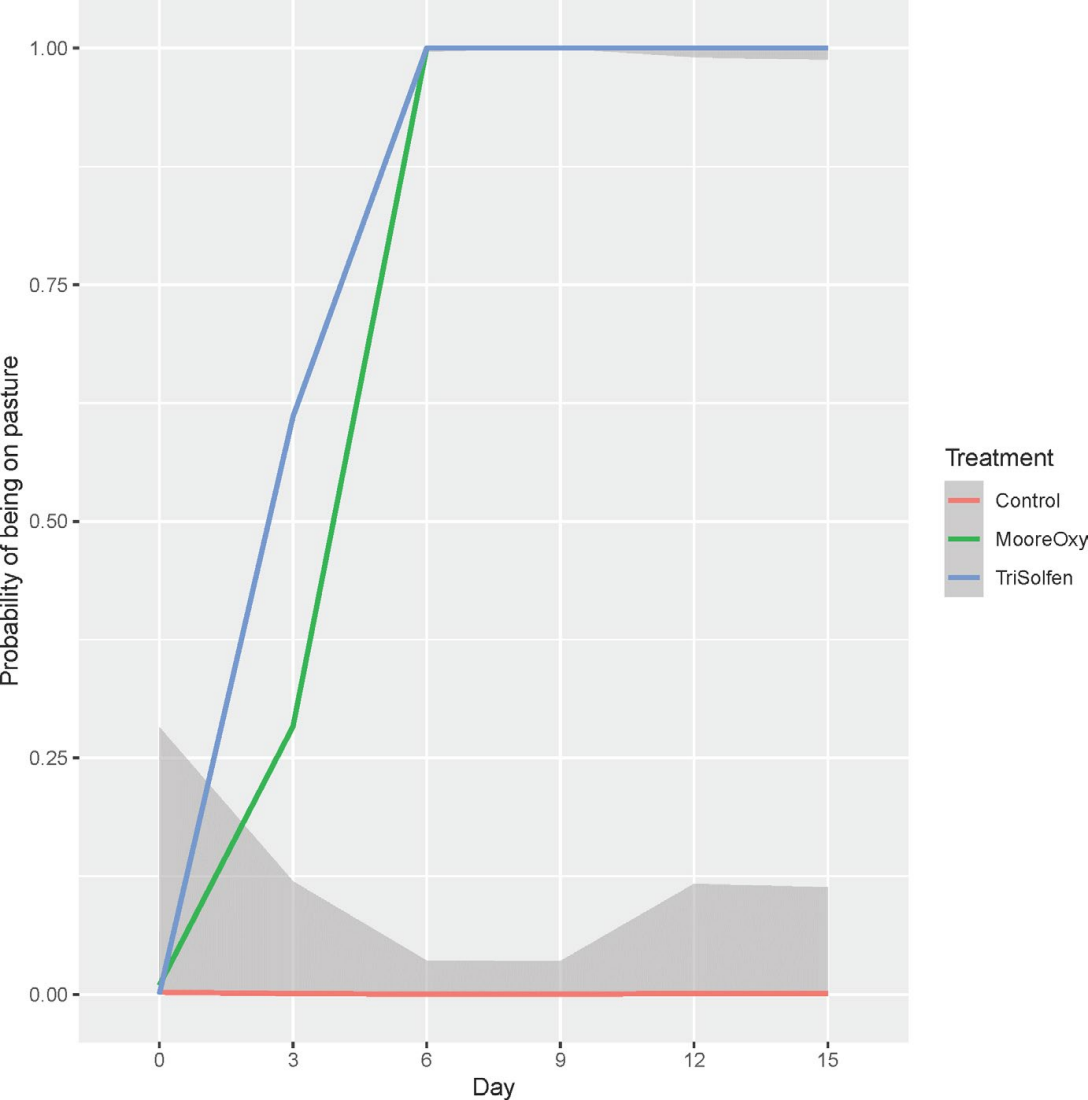
Model‐based probabilities of cattle being on pasture across the six study days, for the three treatment groups. Shaded areas are ±1 *SE* of the mean (where available) [Colour figure can be viewed at wileyonlinelibrary.com]

There was a highly significant Treatment × Day interaction for the presence of cattle accessing pasture (*p* = 1.5 × 10^–6^). It was apparent that Control cattle basically never returned onto pasture during the trial (Figure 6). However, for the two treatment groups, after initially none being on pasture, all were on pasture by Day 6. Although TS showed a faster return to pasture than MO, it was not possible to formally test this. There were no significant effects of Age (*p* = .791) nor Sex (*p* = .661) on the probability of being on pasture; there was some evidence of breed differences (*p* = .023), although breed estimates could not be relied upon because of small number in some breeds.

### 3.6 Salivation

**FIGURE 7:**
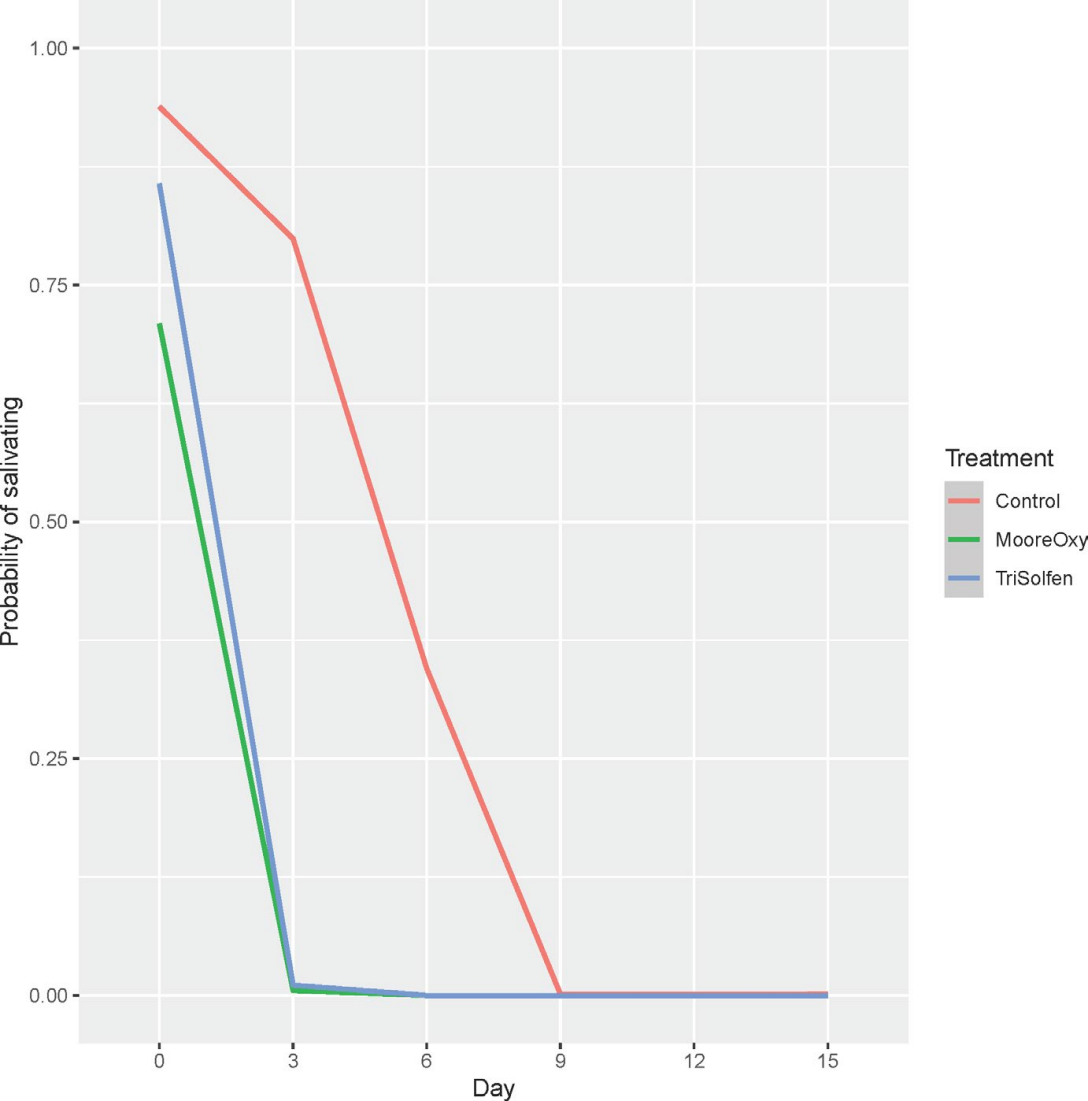
Model‐based probabilities of cattle salivating across the six study days, for the three treatment groups [Colour figure can be viewed at wileyonlinelibrary.com]

There was no significant Treatment × Day interaction for occurrence of salivation (*p* = .496), nor was there an overall main effect of Treatment (*p* = .401). Model‐based probabilities of salivating are shown (Figure 7). However, from initial high rates of salivating, there was a significant decline over the study period (*p* = 6.4 × 10^–12^), with an apparent faster rate of decline for the two treatment groups compared with the control group. There were no significant effects of Age (*p* = .619), Breed (*p* = .096) nor Sex (*p* = 1.000) on instances of salivation.

The results of the analysis to compare the number of days that cattle were salivating are displayed (Table 3). As in the previous analysis, no significant effects of Treatment, nor Age, Breed or Sex were detected (all *p* > .1), with the two treatment groups spending less time salivating.

**TABLE 3:** Analysis of the number of days that cattle are salivating

Variable	*p*‐Value	Control	Moore‐Oxy^®^	Tri‐Solfen^®^
Treatment	.146	1.59^A^ ± 0.82	0.49^A^ ± 0.39	0.56^A^ ± 0.46
Age	.171			
Breed	.537			
Sex	.500			

Note

Means sharing the same superscript alphabets are not significantly different (*p* > .05).

### 3.7 Cost of therapy model

The treatment types and costs for FMD therapy in Cameroon were estimated, enabling a model to support therapy decisions for both individual farmers and public health policy makers (Table 4). With a single treatment of 1ml per lesion for TS, at USD0.50 per ml, the cost of treatment per animal is estimated between USD1.50 and 2.50.

**TABLE 4:** Treatment types used by farmers, application method, days of treatment and estimated daily and total costs

Treatment choice	Application	Est. cost/day (USD)	Treatment days	Est. cost/animal (USD)
Moore‐Oxy	Injection	0.85	3	2.55
Procaine penicillin	Injection	0.17	3	0.51
Oxytet 30%	Injection	0.85	3	2.55
Survidium	Injection	0.85	5	4.25
Insecticide & Petrol	Topical	0.42	7	2.94
Traditional drugs	Topical/oral	0.51	7	3.57
OXYDOZER 50	Injection	0.85	3	2.55
Tri‐Solfen	Topical	/	1	2.50

Note

Moore‐Oxy^®^ has a 7 day milk WHP and 21 meat WHP. Tri‐Solfen^®^ has a recommended 4 day WHP for milk and meat in Lao PDR.

## 4 DISCUSSION

This study reports the first field treatment trial using the novel topical anaesthetic wound formulation (Tri‐Solfen^®^) as a therapy for the clinical management of FMD in Africa. It compared the clinical efficacy of this approach with the commonly used parenteral antibiotic treatment for FMD (Moore‐Oxy^®^) and animals that remained untreated. Despite necessary limitations on numbers of farmers and cattle recruited for the trial, due to low availability of resources, the results obtained provided a clear indication of the therapeutic advantage for TS for FMD, as recently described in Laos (Windsor et al., 2020). Importantly, all participants considered it was very successful trial, with high levels of appreciation by participants for their involvement, the product examined (TS) and the clear clinical responses observed.

Lesion healing scores across the three groups revealed superior results for the TS‐treated cohort, with more rapid healing of coronary band lesions incurred from FMD. Although the MO‐treated cohort also achieved a reasonable score, the untreated control animals had lesions persisting for in excess of 2 weeks, indicative of the prolonged clinical course and debilitating impacts of FD on grazing animals. Although a subjective measurement, these scores provide a useful indication of the healing rate of FMD lesions in field conditions in Cameroon.

Similarly, appetite scores were highest in the TS‐treated cohort, indicating that the treatment supported the rapid return of appetite, presumably reflecting the impact of oral anaesthesia enabling animals to eat shortly after treatment. After 3 days, it appeared that oral pain for the TS‐treated cohort may have returned, although the return of appetite by day 6 suggests more rapid healing of oral lesions than untreated animals, as previously reported at 5 days (Windsor et al., 2020). Superior scores in this group were recorded at each data collection between day 6 and day 15, with the cohort receiving treatment with MO also having reasonable appetite scores between days 6 and 15. The control cohort cattle had poor scores throughout, indicating that appetite remains poor for in excess of 2 weeks when FMD remains untreated. These findings suggest the possibility that secondary bacterial infections may have compromised the healing of oral lesions, with the antiseptic (cetrimide) and low pH properties of TS, and the presence of circulating antibiotic of MO, ameliorating this.

Lesion size was measured (in cm) at six data collection intervals, with the FMD‐infected cattle lesion size decreasing rapidly in the TS‐treated cohort. By day 6, this cohort was performing well with average lesion size at 0.33 cm, despite commencing in the trial with the highest average lesion size. By day 9, the lesions in the TS‐treated cohort had almost entirely disappeared, remaining at 0 cm through day 12 and 15. This is consistent with recent findings that healing of FMD lesions (Windsor et al., 2020) and induced husbandry wounds (Ferrer et al., 2020; Roberts & Windsor, 2019) is enhanced following topical treatment with TS, despite likely depletion of the actives in the formulation. This may be attributable to inclusion of a gel matrix providing prolonged protection for lesion and wound recovery. Interestingly, the average lesion size in the control group increased as the trial progressed, suggesting that healing was compromised, presumably from secondary bacterial infection. This may also explain the observation that in the MO‐treated cohort, the average lesion size decreased until day 12, where it was recorded to increase from 0.17 cm to 0.75 cm at day 15, possibly from the onset of secondary infection presumably following depletion of residual oxytetracyline. However, differences in stage of infection may also have influenced the study here, particularly as the average lesion size in the control cohort was 0cm at trial on day 0, with vesicles yet to rupture.

The farmer observations of clinical impacts also provided interesting results which could be used as animal welfare indicators as well as indicators of clinical response to the treatments. The mobility of all 12 cattle in the TS‐treated cohort returned immediately, suggesting this is a very useful therapy to enable cattle to walk and gain access to water and feed. In the MO‐treated cohort, 10 of the animals also immediately returned to walking. However, of the control group, only five had immediate mobility and four animals did not return to walking even by day 15. There was one animal in the MO‐treated cohort that did not return to mobility until day 12. In total, 7/36 (19.4%) cattle had not returned to mobility by day 9, indicating that FMD is a severe disease as it renders animals immobile and that although FMD is considered a low mortality disease, the animal welfare impacts are clearly considerable.

As oral vesicular lesions can have significant impacts on animal behaviours, recording the number of days the animals were anorexic was considered important. In total, 11/12 cattle in the TS‐treated cohort were reported to be eating the same day as treatment (day 0). This compared to 9/12 cattle in the MO‐treated cohort, with only 2/12 in the control cohort, with 9/12 of these untreated cattle remaining anorexic until day 15. Further, salivation is a common clinical sign with cattle with vesicular disease, considered an overt indicator of oral lesions and presumably oral pain. In all three cohorts, the majority of cattle had ceased salivating at day 0, with the TS‐treated cohort achieving 11/12, MO‐treated cohort 10/12 and the controls 8/12.

Farmer reports of visible lameness of trial cattle indicated that 9/12 cattle in the TS‐treated cohort ceased any lameness at day 0, compared to 5/12 in the MO‐treated cohort and 3/12 in the controls. By day 6, no further cattle in the TS‐treated cohort showed any lameness compared to 5/12 cattle in the MO‐treated cohort still showing lameness by days 12 and 15, with lameness only ceasing by day 15 in 9/12 control cattle. For the question of how many days prior to cattle returning to grazing, mixed results were recorded. In the control cohort, 9/12 were reported grazing by day 0, with 8/12 in the TS‐treated cohort returned to grazing by day 3 and all 12/12 in the MO‐treated cohort returning to grazing by day 6. TS has previously been shown to be efficacious in controlling pain during the surgical treatment of hoof lesions in cattle (Stilwell et al., 2019).

When asked for a broad overview of treatments applied to FMD, six options were provided, including antibiotic formulations, traditional drugs, insecticides and petrol. Without speculating on the therapeutic potential of each treatment, it does indicate that farmers and para‐veterinarians are inclined to treat affected animals with *something*. The data on costs of treatment suggest there are only minimal differences between treatments and that cost should not be an impediment to the changing of treatment choice. TS offers a non‐antimicrobial therapeutic option for treating clinical FMD, one that appears to have superior clinical efficacy to prolonged parenteral use of oxytetracylcine and potentially other antimicrobial therapies. As noted, use of TS potentially reduces the risk of AMR following FMD therapy (Windsor et al., 2020), yet as suggested in these results, may also decrease putative secondary bacterial infections occurring following the rupture of FMD vesicles. Of interest was a recent observation that a lower rate of secondary infection occurred following application of TS during surgical tail‐docking of lambs, enabling the consideration of replacing routine antibiotic cover with a topical anaesthetic and antiseptic wound formulation (Ferrer et al., 2020).

Field studies involving animal treatments with assessments of clinical impacts are challenging and subject to potential bias, reflected in the limited published literature on FMD therapy. However, the results obtained in this study demonstrate the superior clinical efficacy of a single application to FMD lesions of the TS topical anaesthetic wound formulation. The participating farmers reported a 100% appreciation for the product in the treatment of FMD and expressed they were happy to have this product available for use in the region. These findings were consistent with those from a recently reported clinical investigation of TS therapy for FMD in Laos (Windsor et al., 2020) and reports of the use of this product for FMD in other countries in Africa, including Niger, Nigeria and Kenya. It was recently suggested that TS may be viricidal against FMDVs if applied prior to or at the time of lesion rupture, potentially limiting virus transmission during FMD outbreaks (Windsor et al., 2020). TS has a pH of 2.7–2.9 that is potentially sufficient to destroy FMDVs, plus a lidocaine concentration likely to be directly viricidal against FMDVs (Haines et al., 1986). Further, TS has recently been shown to reduce viral load in cutaneous lesions in sheep caused by the Orf virus (PW, unpublished observations). This suggests that adoption of TS therapy for FMD may reduce disease transmission, the extent of animal suffering, plus rural household and national socioeconomic losses in developing countries.

This study in Cameroon provides the quantitative assessment confirming that TS is efficacious in hastening clinical recoveries, immediately addressing pain and invoking more rapid healing of FMD lesions, as observed qualitatively in Laos (Windsor et al., 2020). As clinical observations of improved animal welfare and enthusiastic feedback from farmers following TS therapy in Laos were confirmed in Cameroon, authorities proceeded with registration of the product in Laos, with registration in Cameroon and several other countries pending. These studies demonstrate that large ruminants affected by FMD and treated with TS exhibit reduced pain, with reductions in both time to recovery and negative productivity impacts. TS therapy imposes no additional financial burden on farmers and has the potential to replace antibiotics for treatment of a viral disease, reducing risks of AMR and residues in the food chain. As farmers in developing countries prioritize therapeutic interventions in FMD outbreaks, TS may also increase the likelihood of presentation of animals for treatment, potentially assisting attempts to improve disease reporting, surveillance and vaccination and biosecurity awareness through extension advice. It is concluded that efforts to promote this new therapeutic approach to FMD management should be supported.

## CONFLICT OF INTEREST

The authors report no conflicts of interest in this work. Studies evaluating Tri‐Solfen® and other therapies for aversive animal husbandry interventions occurring prior to this study were funded by an Australian Research Council Linkage Grant from the Australian government with financial contributions from Animal Ethics Pty Ltd Australia and Bayer Animal Health Australia. However, this current study did not receive funding from either of these companies, nor did they have a role in study design, data collection and analysis, decision to publish or preparation of the manuscript.

## AUTHOR CONTRIBUTIONS

PW and SL designed the study, and PW, JY and PT provided analytical and writing support. SL with support from JM, KB, KA and SD, performed the field trial activities. PT conducted the statistical analyses and all authors contributed to the final draft of the manuscript.

## ETHICAL APPROVAL

The authors confirm that the ethical policies of the journal as noted on the authors guidelines page have been adhered to. In addition to following current procedures on animal and human ethics processes in Cameroon, the authors communicated with their Australian collaborators to ensure they complied with the National Health and Medical Research Council's (NHMRC) National Statement on Ethical Conduct in Human Research (2007) and the Universities Australia Australian Code for the Responsible Conduct of Research. This included ensuring that all participants provided verbal informed consent for the collection of animal blood samples, tissues, farmer interviews and participation in videos and images, where written consent was unavailable due to farmer illiteracy.

## Data Availability

The data that support the findings of this study are available from the corresponding author upon reasonable request.

## References

[tbed13923-bib-0001] Agresti, A. (2002). Categorical data analysis (2nd edn.). John Wiley & Sons.

[tbed13923-bib-0002] AL‐Lethie, A., AL‐Lethie, S. F., El‐Hawari, K., El‐Khabaz, A. S., Elmeligy, E., Khalphallah, A., Usama, T., & Mahmoud, U. T. (2018). Evaluation of clinical recovery and healing of oral lesions by 3 different therapeutic regimens in cattle with foot and mouth disease (FMD). Assiut Veterinary Medicine Journal, 64(156), 89–95.

[tbed13923-bib-0003] Bates, D., Maechler, M., Bolker, B., & Walker, S. (2015). Fitting linear mixed‐effects models using lme4. Journal of Statistical Software, 67(1), 1–48. 10.18637/jss.v067.i01

[tbed13923-bib-0004] Bertram, M. R., Delgado, A., Pauszek, S. J., Smoliga, G. R., Brito, B., Stenfeldt, C., Hartwig, E. J., Jumbo, S. D., Abdoulmoumini, M., Oliva Marie, A. A., Salhine, R., Rodriguez, L. L., Garabed, R., & Arzt, J. (2018). Effect of vaccination on cattle sub‐clinically infected with Foot‐and‐Mouth disease virus in Cameroon. Preventive Veterinary Medicine, 155, 1–10. 29786519 10.1016/j.prevetmed.2018.04.003

[tbed13923-bib-0005] Christensen, R. H. B. (2019). Ordinal: regression models for ordinal data. R package version 2019.12‐10. https://CRAN.R‐project.org/package=ordinal

[tbed13923-bib-0006] Ehizibolo, D. O., Fish, H., Brito, B., Bertram, M. R., Ardo, A. G., Ularamu, H. G., Lazarus, D. D., Wungak, Y. S., Nwosuh, C. I., Smoliga, G. R., Hartwig, E. J., Pauszek, S. J., Dickmu, S., Abdoulkadiri, S., & Arzt, J. (2019). Re‐emergence of the novel topotype of foot and mouth disease virus serotype SAT1 in Nigeria and Cameroon. GFRA 2019 Scientific MeetingBangkok, Thailand, October 29–31.

[tbed13923-bib-0007] Fakhrul‐Islam, K. M., Jalal, M. S., Podder, S., Quader, M. N., Sahidur‐Rahman, M., Dutta, A., & Mazumder, S. (2016). Clinical investigation of foot and mouth disease of cattle in Batiaghata Upazilla veterinary hospital. Bangladesh. Veterinary Sciences: Research and Reviews, 2(3), 76–81. 10.17582/journal.vsrr/2016.2.3.76.81

[tbed13923-bib-0008] FAO (2015). Strategic Plan for the Control of Foot and Mouth Disease in Cameroon. In: *Towards improving the control of transboundary animal diseases of trade livestock*. Cameroon: FAO, OMC. MTF/CMR/034/STF. https://www.standardsfacility.org/information‐session‐cameroon

[tbed13923-bib-0009] Ferrer, L. M., Lacasta, D., Ortín, A., Ramos, J. J., Tejedor, M., Borobia, M., Pérez, M., Castells, E., Ruiz de Arcaute, M., Héctor Ruiz, H., & Windsor, P. A. (2020). Impact of a Topical Anaesthesia Wound Management Formulation on Pain, Inflammation and Reduction of Secondary Infections after Tail Docking in Lambs. Animals, 10, 1255. 10.3390/ani10081255 PMC7459688 32722010

[tbed13923-bib-0010] Frossardm, J., & Renaud, O. (2019). permuco: Permutation tests for regression, (repeated measures) ANOVA/ANCOVA and comparison of signals. R package version 1.1.0. https://CRAN.R‐project.org/package=permuco

[tbed13923-bib-0011] Gakuya, D. W., Mulei, C. M., & Wekesa, S. B. (2011). Use of Ethnoveterinary remedies in the Management of Foot and Mouth Disease lesions in a Dairy Herd. African Journal Traditional and Complementary Alternative Medicine, 8(2), 165–169. 10.4314/ajtcam.v8i2.63204 PMC3252696 22238498

[tbed13923-bib-0012] Haines, H. G., Dickens, C. B., & Brigham, D. P. (1986). Antiviral pharmaceutical preparations and methods for their use. Patent US 4628063A

[tbed13923-bib-0013] Hervé, M. (2020). RVAideMemoire: testing and plotting procedures for biostatistics. R package version 0.9‐77. https://CRAN.R‐project.org/package=RVAideMemoire

[tbed13923-bib-0014] Lendzele, S. S., Abdoulmoumini, M., Marvoungou, M. J., Ikoum, D., Mohammadou, B., Oumarou, L., Hiol, V. D., Rodrigue, M. N., Zinga‐Koumba, C. R., Acapovi‐Yao, G. L., Simon, D., & Garabed, R. (2019). Serological Epidemiology of Foot‐and‐mouth Disease among Sedentary Mixed‐species Herds in Adamawa Region, Cameroon. Journal of Advances in Microbiology, 17(2), 1–14.

[tbed13923-bib-0015] Lendzele, S. S., Marvoungou, M. J., & Rodrigue, M. N. (2019). Veterinary pharmaceuticals sold in cattle markets for the management of foot‐and‐mouth disease and flies in vina division (Adamawa‐Cameroon). Journal of Dairy and Veterinary Science, 10(2), 555782. 10.19080/JDVS.2019.10.5557820010

[tbed13923-bib-0016] Lenth, R. (2020). emmeans: Estimated marginal means, aka least‐squares means. R package version 1.4.7. https://CRAN.R‐project.org/package=emmeans

[tbed13923-bib-0017] Ludi, A., Ahmed, Z., Pomeroy, L. W., Pauszek, S. J., Smoliga, G. R., Moritz, M., & Rodriguez, L. L. (2016). Serotype Diversity of Foot‐and‐Mouth‐Disease Virus in Livestock without History of Vaccination in the Far North Region of Cameroon. Transboundary and Emerging Diseases, 63(1), e27–e38. 10.1111/tbed.12227 24735162 PMC4499489

[tbed13923-bib-0018] MINEPIA (2013). Ministère de l’Elevage, des Pêches et des Industries Animales. MINEPIA Policy Document, 29.

[tbed13923-bib-0019] Misk, N. A., Misk, T. N., & Rateb, H. Z. (2015). Assessment and Topical Treatment of Lesions of Foot and Mouth Disease in Cattle. Assiut Veterinary Medicine Journal, 61, 75–81.

[tbed13923-bib-0020] Roberts, C. D., & Windsor, P. A. (2019). Innovative pain management solutions in animals may provide improved wound pain reduction during debridement in humans: An opinion informed by veterinary literature. International Wound Journal, 16(4), 968–973. 10.1111/iwj.13129 30938098 PMC7948712

[tbed13923-bib-0022] Stilwell, G. T., Ferrador, A. M., Santos, S., Domingues, J. M., & Carolin, N. (2019). Use of topical local anesthetics to control pain during treatment of hoof lesions in dairy cows. Journal of Dairy Science, 102, 6383–6390. 10.3168/jds.2018-15820 31030913

[tbed13923-bib-0024] Windsor, P. A., Earp, F., MacPhillamy, I., Khounsy, S., Young, J., & Bush, R. D. (2020). A new topical therapy for Foot‐and‐mouth disease addresses animal welfare and other issues. Veterinary Medicine: Research Reports, 11, 99–107. 10.2147/VMRR.S273788 PMC7549654 33117659

[tbed13923-bib-0025] Windsor, P. A., Lomax, S., & White, P. (2016). Pain management for improved small ruminant welfare. Small Ruminant Research, 142, 55–57.

